# Involvement of sugar and abscisic acid in the genotype-specific response of rose to far-red light

**DOI:** 10.3389/fpls.2022.929029

**Published:** 2022-07-22

**Authors:** Laurent Crespel, Camille Le Bras, Thomas Amoroso, Bénédicte Dubuc, Sylvie Citerne, Maria-Dolores Perez-Garcia, Soulaiman Sakr

**Affiliations:** ^1^Institut Agro, Université d’Angers, INRAE, IRHS, SFR 4207 QUASAV, Angers, France; ^2^ASTREDHOR, Institut des professionnels du végétal, Paris, France; ^3^Institut Jean-Pierre Bourgin, INRAE, AgroParisTech, Université Paris-Saclay, Versailles, France

**Keywords:** rose, architecture, far-red light, genotype-specific response, sugar, abscisic acid

## Abstract

Plant architecture determines yield (fruit or flowers) and product quality in many horticultural species. It results from growth and branching processes and is dependent on genetic and environmental factors such as light quality. Highly significant genotype and light quality effects and their interaction have been demonstrated on the architecture of rose. Far-red (FR) light is known for its favourable effect on plant growth and development. We evaluated the effect of FR on rose growth and development and its interaction with the genotype through architectural, eco-physiological (net photosynthesis rate) and biochemical (sugar and hormone concentrations) approaches. Two cultivars (‘The Fairy’ – TF – and Knock Out^®^ Radrazz – KO) with contrasting architectures were grown in a climate chamber under FR or in the absence of FR at an average photosynthetic photon flux density (400–700 nm) of 181.7 ± 12.8 μmol m^−2^ s^−1^ for 16 h. A significant effect of FR on the architecture of TF was demonstrated, marked by greater stem elongation, shoot branching and flowering, while KO remained insensitive to FR, supporting a genotype x FR interaction. The response of TF to FR was associated with improved photosynthetic capabilities, while KO exhibited an elevated level of abscisic acid (ABA) in its leaves. FR-dependent ABA accumulation might inhibit photosynthesis and prevent the increased plant carbon status required for growth. From a practical perspective, these findings argue in favour of a better reasoning of the choice of the cultivars grown in lighted production systems. Further investigations will be necessary to better understand these genotype-specific responses to FR and to unravel their molecular determinants.

## Introduction

Plant architecture—e.g. the number of branches—determines yield (whether fruit or flowers) in many horticultural species (Bredmose, 1993; Liu et al., 2021). It also defines the visual quality of potted ornamental plants by determining their shape (Boumaza et al., 2009; Garbez et al., 2015). Plant architecture is the result of the positioning of aerial organs in space according to the specific organisation rules. It is a complex trait when it comes to analysing it, particularly in ornamental shrubs, as it involves a diversity of axes and several orders of branching, as observed in rose, camellia or rough-leaved hydrangea (Morel et al., 2009; Galopin et al., 2011; Crespel et al., 2012). In order to facilitate its analysis, we developed a phenotyping method by 3D digitalisation of rose, chosen for its ornamental economic importance (Gudin, 2000). This method consists in structuring the plant architecture into axes and metamers, and characterising them at the morphological (diameter and length), topological (succession and branching relationships) and geometrical (organization in space) levels (Morel et al., 2009; Crespel et al., 2012, 2013). All these efforts laid the foundation for an almost exhaustive description of the plant architecture at different scales (plant, axis and metamer).

Plant architecture depends on both genetic and environmental factors. Most of the architectural characteristics are moderately to strongly heritable (Li-Marchetti et al., 2017; Ye et al., 2017; Jiji Allen et al., 2019). In rose, some of them (e.g., the length and inclination of the axes) are weakly heritable and strongly influenced by environmental factors such as water supply and light intensity (Demotes-Mainard et al., 2013; Li-Marchetti et al., 2015, 2017). Thus, plant architecture can be genetically controlled through varietal improvement and manipulation of the environmental conditions of plant cultivation, such as water restriction (Demotes-Mainard et al., 2013; Li-Marchetti et al., 2015), mechanical stimulation (Morel et al., 2012) or modified light quality (Rajapakse and Kelly, 1993). The effect of these cultivation techniques has mostly been evaluated on one cultivar, without taking the variability of the genotypic response into account, i.e., the genotype x environment interaction, as often observed in rose (Crespel et al., 2014; Li-Marchetti et al., 2015, 2017).

Several families of photoreceptors allow the plant to perceive changes in the light quality and adapt its development. Red light—red (R; 600–700 nm) and far-red (FR; 700–800 nm)—is perceived by phytochromes, while blue light (B; 400–500 nm) is perceived by several families of photoreceptors, particularly cryptochromes (Paradiso and Proietti, 2021). Elongation, branching and flowering are particularly influenced by red and blue lights (Paradiso and Proietti, 2021). For example, a decreased R/FR ratio led to increased plant height—a shade avoidance syndrome—in foxglove (Elkins and van Iersel, 2020) or accelerated flowering in snapdragon (Park and Runkle, 2017), while increased B-light intensity inhibited stem elongation in radish and soybean (Cope and Bugbee, 2013).

Photoselective plastic films that intercept FR and increase the R/FR ratio were first used by horticulturists to modify light quality (Rajapakse et al., 1999; Cerny et al., 2003; Li et al., 2003). More recently, the development of light-emitting diodes (LEDs) made it possible to widely manipulate the light spectrum: other ratios of wavelengths (B/R, B/Green (G), etc.) became available, opening up possibilities to modify the plant architecture, as observed in many horticultural species (Paradiso and Proietti, 2021).

We tested several light modalities to control the architecture of rose, by differentiating B/R (0.1–1.0 ratios) and R/FR (7.5–23.2 ratios) instead of the sole wavelengths (Crespel et al., 2020). A significant light quality effect was shown under FR enrichment (the lowest B/R and R/FR ratios), leading to elongated axes and an elevated number of axes, including flowering axes. These light-mediated architectural modifications, i.e., etiolated plants and accelerated flowering, have been reported in tomato grown under FR (Kalaitzoglou et al., 2019; Zhang et al., 2019). They might result from better light interception by the plant, and thereby increased photosynthesis. The light quality also regulates the plant architecture *via* hormones. *Arabidopsis thaliana* and sunflower displayed elevated levels of auxins and gibberellins in their internodes and abscisic acid (ABA) in their buds in response to FR, resulting in axis elongation and branching inhibition, respectively (Kurepin et al., 2007, 2012; Reddy et al., 2013). However, it was difficult to attribute the observed effect to the sole FR in our previous study because the two B/R and R/FR wavelength ratios were modified simultaneously (Crespel et al., 2020).

As previously observed for other environmental factors, like water supply and light intensity (Crespel et al., 2014; Li-Marchetti et al., 2015, 2017), the effect of light quality on rose architecture is genotype dependent (Crespel et al., 2020). The basic mechanisms behind this genotype-specific response are still unknown. A strong genotype x light quality interaction characterised by contrasting genotypic responses has been reported in rose, while only a limited one has been described in chrysanthemum (Dierck et al., 2017). This situation underlines that rose is a powerful plant model to investigate the basic mechanism orchestrating this genotype x light quality interaction. All these findings prompted us to set up a stepwise and multi-disciplinary (architectural, eco-physiological and biochemical) approach to better understand the effect of FR on the plant architecture and its interaction with the genotype. Two rose varieties (‘The Fairy’ and Knock Out® Radrazz) with highly contrasting responses to light quality were selected and grown under FR or in the absence of FR. Our data underline for the first time the role of two endogenous factors—sugar and ABA—in the genotype-specific response to FR.

## Materials and methods

This study was carried out in Angers (France), in the experimental facilities of the Research Institute on Horticulture and Seeds.

### Plant material

The plant material was composed of two rose cultivars with contrasting shapes: ‘The Fairy’ (TF; ground cover), chosen for its architectural plasticity to light quality, and Knock Out® Radrazz (KO; upright bush), chosen for its insensitivity to light quality (Abidi et al., 2013; Crespel et al., 2020).

The plants were obtained from cuttings taken from mother plants grown in pots in the greenhouse. The cuttings were composed of a single metamer taken from the median zone of the stems. The cuttings were planted in plugs (35 mm diameter and 40 mm height), composed of a non-woven cloth containing a mixture of fine peat and perlite. The rooting phase took place in a glass greenhouse, under a plastic tunnel. The mean temperature was 22°C during the day and 18°C at night, and relative humidity was maintained at saturation by a fine mist humidifier.

The young plants were potted 4 weeks later in 0.5 l pots, in a substrate composed of peat (50%), perlite (40%) and coconut fibre (10%), and then acclimatised in a greenhouse for 1 week.

### Experimental conditions

**Figure 1:**
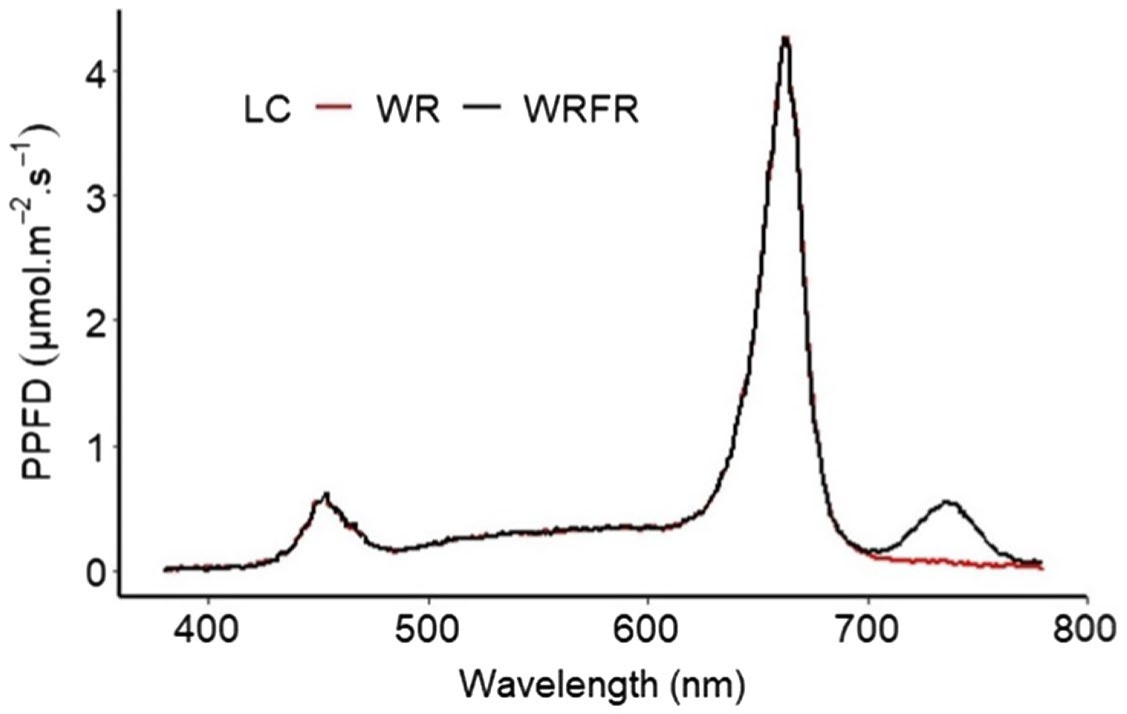
Spectral distribution of two light conditions (LC; WR and WRFR) provided by white (W), red (R), and far-red (FR) light-emitting diodes (LEDs).

After the acclimatisation phase, the plants were grown in a climate chamber composed of two shelves (3.80 m × 0.80 m), spaced 85 cm apart. About 30 plants of each cultivar were randomly placed on each shelf. Mineral nutrition was provided by fertirrigation, in subirrigation, with a balanced liquid fertiliser (N-P_2_O_5_-K_2_O) 3–2–6, a pH of 6.5 and an average electrical conductivity of 1.2 mS.cm^−1^. The air temperature was maintained at 20°C during the day and 18°C at night, with a relative humidity of 70%. The climatic chamber was continuously ventilated. The CO_2_ concentration corresponded to that of the atmosphere. The plants were subjected to a photophase of 16 h, with an average photosynthetic photon flux density (PPFD; 400–700 nm) of 181.7 ± 12.8 μmol m^−2^ s^−1^ (a daily light integral of 10.5 mol m^−2^ d^−1^), provided by a LED lighting device composed of panels of 12 white, red and far-red LED tubes (Cesbron, Saint Sylvain d’Anjou, France) according to the tested light condition. The climate chamber was divided into two compartments to test two light conditions simultaneously, with the following spectral characteristics: the light spectrum of condition 1 (WR) was composed of 22.2 μmol m^−2^ s^−1^ of B (or 12.1% of the PPFD; 400–500 nm), 31.3 μmol m^−2^ s^−1^ of G (17.1%; 500–600 nm), 129.8 μmol m^−2^ s^−1^ of R (70.8%; 600–700 nm) and 4.7 μmol m^−2^ s^−1^ of FR (700–800 nm). The light spectrum of condition 2 (WRFR) was composed of 21.3 μmol m^−2^ s^−1^ of B (11.8%), 29.8 μmol m^−2^ s^−1^ of G (16.5%), 129.0 μmol m^−2^ s^−1^ of R (71.7%) and 20.9 μmol m^−2^ s^−1^ of FR (Figure 1). The LED lighting device was positioned at 65 cm from the top of the pot.

The PPFD and light spectrum were measured using a Rainbow Light MR16 spectrophotometer (Rainbow Light Technology CO., LTD, Taiwan) placed at the top of pot. The spectral characteristics (PPFD, TPFD, YPFD, B/R, B/G and R/FR) of each condition are specified in Table 1. No significant difference in PPFD, TPFD and YPFD was observed between the two light conditions (Table 1).

**Table 1:** Spectral characteristics of the two light conditions WR and WRFR.

Light conditions	PPFD (μmol m^−2^ s^−1^)	TPFD (μmol m^−2^ s^−1^)	YPFD (μmol m^−2^ s^−1^)	B/R	B/G	R/FR
WR	183.3a^1^	188.0a	164.8a	0.2a	0.7a	27.7b
WRFR	180.1a	201.1a	164.1a	0.2a	0.7a	6.2a

PPFD, photosynthetic photon flux density (400–700 nm); TPFD, total photon flux density (400–800 nm); YPFD, yield photon flux density, i.e., the product of TPFD and relative quantum efficiency; B/R, blue light (400–500 nm) over red light (600–700 nm) ratio; B/G, blue light (400–500 nm) over green light (500–600 nm) ratio; R/FR, red light (600–700 nm) over far-red light (700–800 nm) ratio.

1 Means followed by the same lowercase letter in the same column are not significantly different (Kruskal & Wallis test, *p* < 0.05).

### Architectural and morphological description of the plant at the ‘visible floral bud (VFB) stage of the order 2 axes carried by the order 1 long axis in apical position’

The plant architecture is characterised by two components: the axis and the metamer. One metamer is composed of one internode, one node, one axillary bud and one leaf, as illustrated by Crespel et al. (2020). These components have topological (succession or branching) relationships between them. The architectural analysis was carried out at two observation scales: the plant scale and the axis scale, using a 3D MicroScribe digitiser (Solution Technologies, Oella, MD, United States). The measurements were recorded in an Excel spreadsheet to build an architectural database. Using this data and a specially developed R-script, the following architectural variables were extracted (Table 2):

– at the plant scale, the total number of axes and determined axes (i.e., flowering axes), according to the order of branching, as well as the number of order 2 axes, according to their position on the order 1 long axis: apical, median and basal.
– at the axis scale, the length of the long axes (comprising five or more metamers; Morel et al., 2009), according to the order of branching.

**Table 2:** List of the architectural variables measured at the ‘VFB of the order 2 axes carried by the order 1 long axis in apical position’ stage, according to three pre-defined categories (elongation, branching and flowering).

Variables	Code
Elongation	
Length of the long axes	LLA
Length of the order 1 long axis	LLA1
Length of the order 2 long axes	LLA2
Branching	
Number of axes	NbA
Number of order 2 axes	NbA2
Number of order 2 axes carried by the order 1 long axis in apical position	NbA2_top
Number of order 2 axes carried by the order 1 long axis in median position	NbA2_med
Number of order 2 axes carried by the order 1 long axis in basal position	NbA2_bas
Number of order 3 axes	NbA3
Flowering	
Number of determined axes	NbDetA
Number of determined order 2 axes	NbDetA2

Three categories of variables were measured at the plant and axis scales: elongation variables (the length and number of metamers that make up a long axis, etc.), branching variables (total number of axes, etc.) and flowering variables (number of determined axes, etc.).

Leaf variables were also measured. Leaves were collected and scanned, placed in an oven at 70°C for 48 h and weighed. The same method was followed for the stems. Using the scans, the total leaf area per plant (tLfarea) was determined, using an ImageJ open-source image analysis software program (National Institutes of Health, United States). The leaf area per leaf (Lfarea) and the specific leaf area (SLA) were deduced by dividing the total leaf area by the number of leaves and the leaf dry weight, respectively.

The measurements were carried out on eight plants per cultivar and light condition, taken from 20 plants in the centre of the shelf, at the ‘VFB stage of the order 2 axes carried by the order 1 long axis in apical position’. Two replicates of the experiment were performed.

### Kinetics of the construction of the order 1 long axis: from elongation to the emission of metamers and branches

To better understand the effect of light quality on the plant architecture observed at the ‘VFB stage of the order 2 axes carried by the order 1 long axis in apical position’, the elongation kinetics of the order 1 long axis and the emission of metamers and branches were studied by digitising the order 1 axis every three or four days (1st, 5th, 8th, 12th, 15th, 19th, 22nd and 26th days of cultivation) until its growth stopped or until the 26th day of cultivation. Using the digitisation measurements, the following variables were extracted: length (LLA1), number of metamers (NbMetA1) and angle of inclination of the cord (relative to the vertical; AngA1) of the order 1 long axis, number of order 2 axes carried by the order 1 long axis in apical position (NbA2_top), length (LLf) and angle of inclination of the cord (relative to the vertical; AngLf) of the 7th leaf carried by the order 1 long axis. The width of the flower bud of the order 1 long axis (WFB) was also measured on the 19th day of cultivation, using photographs and ImageJ software.

The measurements were carried out on 16 plants per cultivar and light condition, taken from the centre of the shelf and monitored throughout the experiment. The experiment was carried out in triplicate.

### Chlorophyll index and net photosynthesis rate measurements

The chlorophyll index of the upper surface of one fully developed apical leaf was measured, using a Dualex optical sensor (Force-A, Paris, France), which determines a chlorophyll index as follows:

Chlorophyll Index $=\frac{lighttransmittedin\;far-red-lighttransmittedin\;red}{lighttransmittedin\;red}$

The net photosynthesis rate (A), the stomatal conductance (g_s_) and intercellular CO_2_ concentration (C_i_) of each leaf chosen for the chlorophyll concentration measurement were also measured 5–8 h after the beginning of the lighting period, using a gas exchange analyser LI-6400 XT (LI-COR, Lincoln, NE, United States), equipped with a transparent leaf chamber. Inside the flow cell, the CO_2_ concentration was 400 μmol mol^−1^, the temperature was 20°C, the air flow was 500 μmol s^−1^ and relative humidity was 65%.

The intrinsic water use efficiency (WUEi) and the instantaneous carboxylation efficiency (A/C_i_) were calculated by dividing the net photosynthesis rate by the stomatal conductance and intercellular CO_2_ concentration, respectively.

The measurements were carried out on eight plants per cultivar and light condition, taken from 20 plants in the centre of the shelf, at the ‘VFB stage of the order 2 axes carried by the order 1 long axis in apical position’. The experiment was carried out in duplicate.

### Sugar and hormone concentration measurements

The sugar concentration in the stem and leaves of the order 1 long axis was determined from eight plants taken from the centre of the shelf on the 19th day of cultivation, at the end of the exponential growth phase of the order 1 long axis, when all the measured architectural variables were impacted by FR. The samples were taken 5–8 h after the beginning of the lighting period and were immediately frozen in liquid nitrogen, and then stored at −80°C. Then, they were freeze-dried and ground. The determination of the sugar concentration was carried out as described by Corot et al. (2017). For each sample, 17 mg of dry powder was homogenised with 1.3 ml of 80% aqueous ethanol at 80°C for 30 min, then 700 μl of 50% aqueous ethanol at 80°C for 30 min. After centrifugation at 11,000 rpm for 5 min at 4°C, the supernatant and the pellet were used for the determination of soluble sugar (sucrose, glucose and fructose) and starch concentration, respectively.

The supernatant was collected and the solvent was removed using a SpeedVac concentrator. The remaining pellet was re-suspended in 0.6 ml water and used for the determination of sucrose, D-glucose and D-fructose concentration, using a Thermo Scientific™ Gallery™ photometric analyser (Thermo Fisher Scientific, Waltham, MA, United States) and ENZYTEC™ fluid kits (R-Biopharm, Darmstadt, Germany).

The pellet was heated for 1 night to 60°C and then re-suspended with 0.2 ml of 80% aqueous ethanol. The starch in the sample was then hydrolysed as follows. The sample was gelatinised by heat treatment at 100°C for 6 min in the presence of 2.9 ml of MOPS sodium buffer and 0.1 ml of thermostable *α*-amylase (3,000 U ml^−1^). After cooling to 50°C, the sample was hydrolysed for 30 min with 4 ml of sodium acetate (200 mM, pH 4.5) and 0.2 ml of amyloglucosidase (200 U ml^−1^). 2 ml of supernatant was centrifuged at 10,000 rpm for 10 min at 4°C. 600 μl was used for the determination of D-glucose concentration, as described previously.

The hormone concentrations (indole-3-acetic acid (IAA), ABA, riboside zeatin, isopentenyladenosine, salicylic acid and jasmonic acid) in the stem and leaves of the order 1 long axis were also determined on the same samples, as described by Li-Marchetti et al. (2015). For each sample, 6.5 mg of dry powder was extracted with 0.8 ml of acetone/water/acetic acid (80/19/1, v:v:v). IAA, ABA, cytokinins, salicylic acid and jasmonic acid stable labelled isotopes used as internal standards were prepared, as described in Le Roux et al. (2014). 1 ng of each and 0.5 ng cytokinins standard were added to the sample. The extract was vigorously shaken for 1 min, sonicated for 1 min at 25 Hz, shaken for 10 min at 10°C and then centrifuged (8,000 g, 10°C, 10 min). The supernatants were collected and the pellets were re-extracted twice with 0.4 ml of the same extraction solution, then vigorously shaken (1 min) and sonicated (1 min, 25 Hz). After the centrifugations, the three supernatants were pooled and dried.

Each dry extract was dissolved in 100 μl of acetonitrile/water (50/50, v/v), filtered and analysed using a Waters Acquity ultra performance liquid chromatograph coupled to a Waters Xevo Triple quadrupole mass spectrometer TQS (UPLC-ESI-MS/MS). The compounds were separated on a reverse-phase column (Uptisphere C18 UP3HDO, 100*2.1 mm*3 μm particle size; Interchim, Montluçon, France) using a flow rate of 0.4 ml min^−1^ and a binary gradient: (A) acetic acid 0.1% in water (v/v) and (B) acetonitrile with 0.1% acetic acid; the column temperature was 40°C. For IAA, ABA, salicylic acid and jasmonic acid, we used the following binary gradient (time, % A): 0 min, 98%; 3 min, 70%; 7.5 min, 50%; 8.5 min, 5%; 9.6 min, 0%; 13.2 min, 98% and 15.7 min, 98% and for cytokinins (time, % A): 0 min, 95%; 13 min, 40%; 16 min, 0% and 16.5 min, 95%.

Mass spectrometry was conducted in electrospray and Multiple Reaction Monitoring scanning mode (MRM mode), in positive ion mode for IAA and in negative ion mode for the other hormones. Relevant instrumental parameters were set as follows: capillary 1.5 kV (negative mode), source block and de-solvation gas temperatures 130°C and 500°C, respectively. Nitrogen was used to assist the cone and de-solvation (150 l h^−1^ and 800 l h^−1^, respectively), argon was used as the collision gas at a flow of 0.18 ml min^−1^. The experiment was carried out in triplicate.

### Data analysis

For each cultivar, the effect of FR was evaluated for all architectural, morphological and eco-physiological variables measured at the VFB stage of the order 2 axes carried by the order 1 long axis in apical position, as well as for the biochemical variables (sugar and hormone concentrations) measured on the 19th day of cultivation. A mixed linear model was used, with the replication of the experiment as a random factor, for a probability *p* < 0.05:

*P_ijk_* = *μ* + *G_i_* + *L_j_* + (*G* × *L*)*_ij_* + *r_k_* + *e_ijk_*

where *P* is the phenotypic value of genotype *i*, for light spectrum *j* and replicate *k*; *μ* is the mean for all genotypes, light spectra and replicates; *G* is the fixed effect of genotype *i*; *L* is the fixed effect of light spectrum *j*; *G* x *L* is the fixed effect of their interaction; *r* is the random effect of the experimental replicate *k* and *e* is the residual error.

The model was estimated using the maximum likelihood (ML) method. Then, a *post-hoc* comparison of means (Tukey’s test) was performed, using the adjusted means (least-square means), for a probability *p* < 0.05. Statistical analyses were carried out using the lme4 and multcomp packages in R (R Foundation for Statistical Computing, Vienna, Austria).

For the architectural variables measured throughout the construction of the order 1 axis, this approach was also applied for each day of cultivation.

## Results

### Architectural and morphological analysis

**Figure 2:**
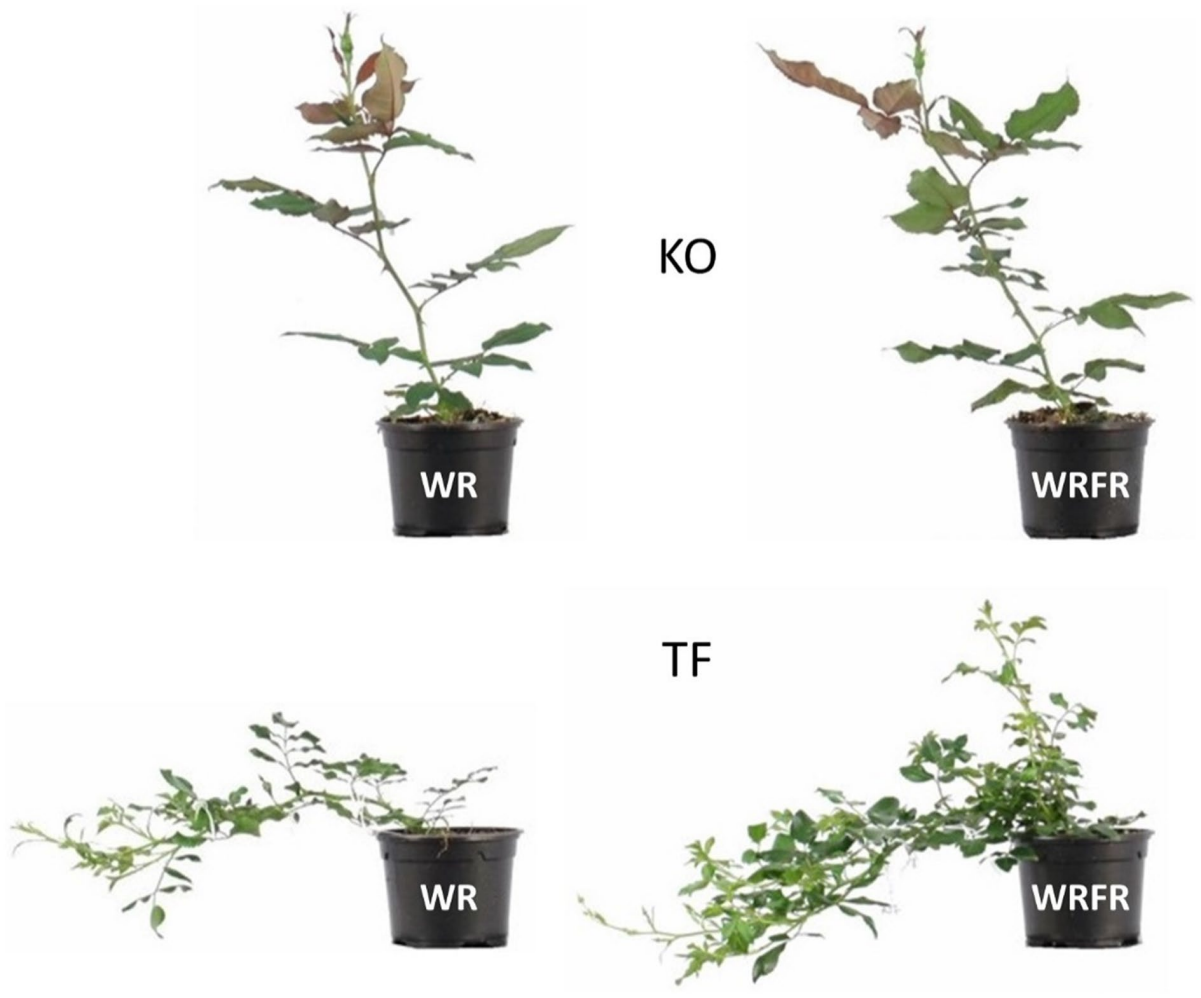
Overall shape of ‘The Fairy’ (TF) and Knock Out^®^ Radrazz (KO) grown in a climatic chamber under two light conditions—WR and WRFR—up to the ‘VFB of the order 2 axes carried by the order 1 long axis in apical position’ stage.

TF and KO responded very differently to FR. At architectural and morphological level, a significant effect of FR was observed in TF for most variables measured both at the ‘VFB of the order 2 axes carried by the order 1 long axis in apical position’ stage or during the construction of the order 1 long axis, while no effect was not displayed in KO; it remained unresponsive to FR (Figure 2; Tables 3, 4).

**Table 3:** Least-square means (LS means) of elongation, branching and flowering variables measured at the ‘VFB of the order 2 axes carried by the order 1 long axis in apical position’ stage for Knock Out^®^ Radrazz (KO) and ‘The Fairy’ (TF) grown in a climate chamber under two light conditions: WR and WRFR.

Genotype	LLA (mm)		LLA1 (mm)		LLA2 (mm)	
	WR	WRFR	WR	WRFR	WR	WRFR
KO	145.4a^1^	152.1a	216.0a	230.6a	130.5a	131.7a
TF	97.1a	152.2b	451.4a	477.0b	53.9a	96.8b
Genotype	NbA		NbA2		NbA3	
	WR	WRFR	WR	WRFR	WR	WRFR
KO	7.1a	8.7a	2.4a	2.5a	2.8a	4.1a
TF	19.4a	24.2b	16.1a	18.0b	0.0a	3.5b
Genotype	NbA2_bas		NbA2_med		NbA2_top	
	WR	WRFR	WR	WRFR	WR	WRFR
KO	0.0a	0.0a	0.0a	0.0a	2.4a	2.4a
TF	1.5a	1.3a	8.1a	8.3a	6.7a	8.4b
Genotype	NbDetA		NbDetA2			
	WR	WRFR	WR	WRFR		
KO	3.3a	3.9a	1.6a	1.5a		
TF	3.3a	10.7b	3.1a	8.1b		

LLA, length of the long axes; LLA1, length of the order 1 long axis; LLA2, length of the order 2 long axes; NbA, number of axes; NbA2, number of order 2 axes; NbA3, number of order 3 axes; NbA2_bas, number of order 2 axes carried by the order 1 long axis in basal position; NbA2_med, number of order 2 axes carried by the order 1 long axis in median position; NbA2_top, number of order 2 axes carried by the order 1 long axis in apical position. NbDetA, number of determined axes; NbDetA2, number of determined order 2 axes.

1 Means followed by the same lowercase letter in the same line are not significantly different (Tukey’s test, *p* < 0.05).

**Table 4:** Least-square means (LS means) of stem and leaf morphological variables measured at the ‘VFB of the order 2 axes carried by the order 1 long axis in apical position’ stage for Knock Out^®^ Radrazz (KO) and ‘The Fairy’ (TF) grown in a climate chamber under two light conditions: WR and WRFR.

Genotype	Stem dry weight (g)		Leaf dry weight (g)			
	WR	WRFR	WR	WRFR		
KO	1.8a^1^	2.1b	2.4a	2.3a		
TF	1.5a	1.9b	2.0a	2.5b		
Genotype	tLfarea (cm^2^/plant)		Lfarea (cm^2^/leaf)		SLA (cm^2^ g^−1^)
	WR	WRFR	WR	WRFR	WR	WRFR
KO	509.3a	490.7a	35.6a	39.4a	220.3a	213.3a
TF	495.1a	649.9b	12.7a	13.2a	244.8a	263.4a

tLfarea, total leaf area; Lfarea, leaf area; SLA, specific leaf area.

1 Means followed by the same lowercase letter in the same line are not significantly different (Tukey’s test, *p* < 0.05).

**Figure 3:**
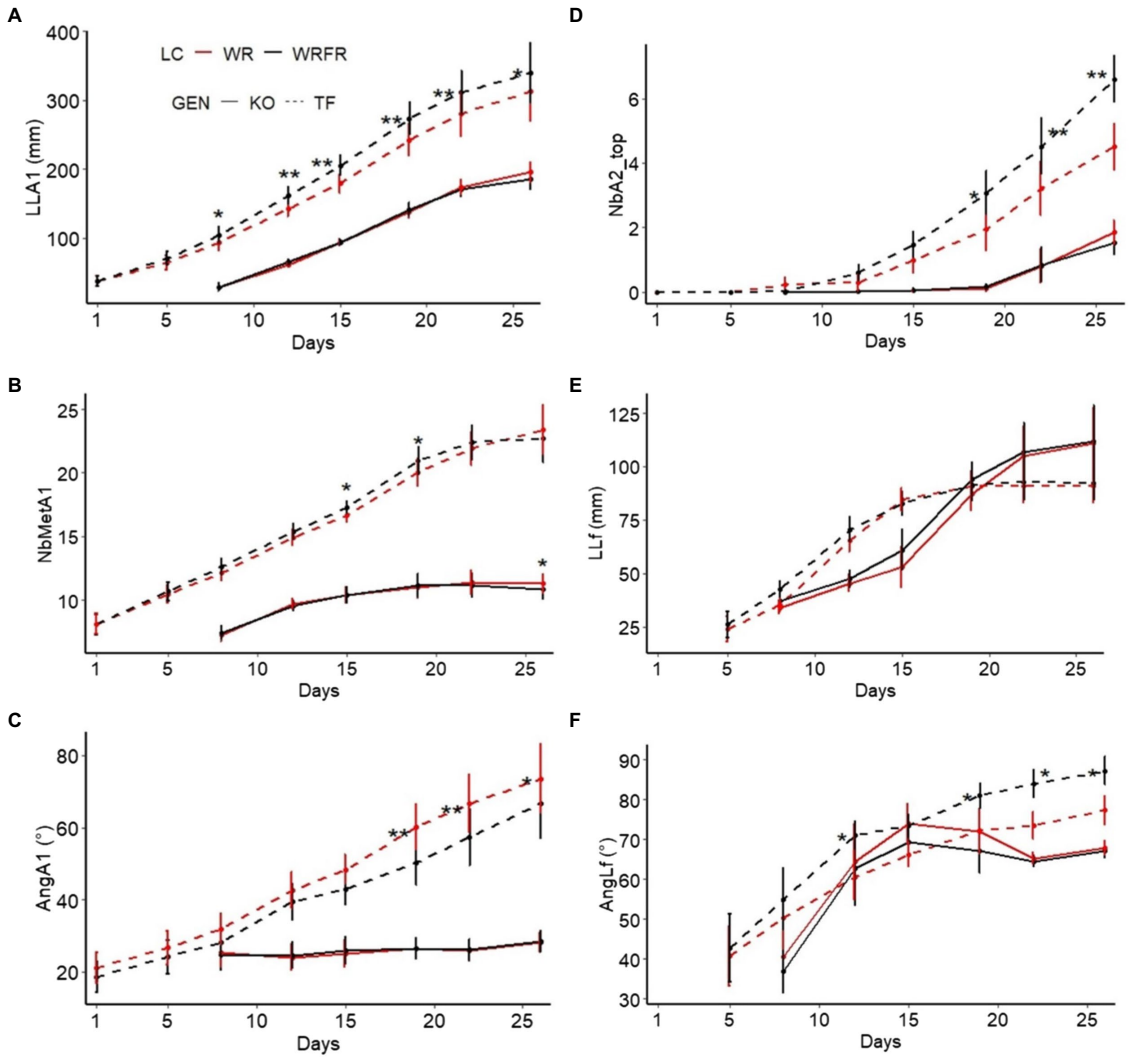
Kinetics of the construction of the order 1 long axis for Knock Out^®^ Radrazz (KO) and ‘The Fairy’ (TF) grown in a climate chamber under two light conditions: WR and WRFR. Kinetics of the elongation of the order 1 long axis (LLA1, length of the order 1 long axis; **A**). Kinetics of metamer emission by the order long 1 axis (NbMetA1, number of metamers of the order 1 long axis; **B**). Kinetics of the inclination angle of the order 1 long axis (AngA1, angle of inclination of the cord (relative to the vertical) of the order 1 long axis; **C**). Kinetics of top branch emission by the order 1 long axis (NbA2_top, number of order 2 axes carried by the order 1 long axis in apical position; **D**). Kinetics of the elongation of the 7th leaf (LLf, length of the 7th leaf carried by the order 1 long axis; **E**). Kinetics of the inclination angle of the 7th leaf (AngLf, angle of inclination of the cord (relative to the vertical) of the 7th leaf carried by the order 1 long axis; **F**). Error bars represent SEMs (*n* = 16). For each genotype (GEN), asterisks indicate statistically significant differences between the light conditions (LC; Tukey’s test, ** *p* < 0.001; * *p* < 0.05).

Under FR, TF plants were etiolated, with longer long axes (LLA; +56.7%), whatever the order of branching (Table 3). The lengthening of the long axes was mostly visible at order 2 (LLA2), with an increase of 79.6%. This etiolating effect of FR appeared very early in plant development: the order 1 long axis was longer from the 8th day of cultivation (LLA1; +12.1%; Figure 3A) and more orthotropic from the 19th day of cultivation (AngA1; −16.4%; Figure 3C). However, the lengthening of the order 1 axis was not associated with an increase in the number of metamers (NbMetA1), except between the 15th (+3.7%) and 19th (+4.5%) day of cultivation (Figure 3B). Plants were also more branched, with a higher number of axes (NbA; +24.7%), whatever the order of branching (Table 3). This increase was located in apical position (NbA2_top; +25.4%; Table 3) and was visible from the 19th day of cultivation (+56.7%; Figure 3D). Under FR, flowering was more abundant, with a higher number of determined (NbDetA; +224.2%) and determined order 2 axes (NbDetA2; +161.3%; Table 3). The flower bud of the order 1 long axis was also more developed, with a higher width on the 19th day of cultivation (WFB; 1.82 mm for WRFR *vs* 1.19 mm for WR, or an increase of 52.9%). These FR-mediated architectural changes were associated with an increase in stem (+26.7%) and leaf (+25.0%) dry biomass (Table 4). Total leaf area was 31.3% higher (tLfarea; Table 4). However, no significant effect of FR was observed on leaf length (LLf), leaf area (per leaf; Lfarea) or specific leaf area (SLA; Figure 3E; Table 4). Only the leaf inclination angle changed, with leaves more plagiotropic, as early as the 12th day of cultivation (AngLf; +17.5%; Figure 3F).

No significant effect of FR was observed on the architecture and the morphology of KO, except for the stem dry weight, with an increase of 16.7% (Figure 3; Tables 3, 4).

### Eco-physiological and biochemical analysis

Eco-physiological and biochemical measurements were performed in order to understand the basic mechanisms of this differential genotypic response.

At eco-physiological level, a significant effect of FR was displayed in TF, with a decrease in chlorophyll index (−11.6%) and an increase in net photosynthesis rate (A; +8.9%), while a decrease in chlorophyll index was displayed in KO (−4.4%; Table 5). For stomatal conductance (g_s_) and intercellular CO_2_ concentration (C_i_), no significant effect of FR was observed, whatever cultivar (Table 5). The same was true for the intrinsic water use efficiency (WUEi; Table 5). However, a significant effect of FR was displayed in TF for instantaneous carboxylation efficiency (A/C_i_), with an increase of 11.3%, while no effect was not observed in KO (Table 5).

**Table 5:** Least-square means (LS means) of the chlorophyll (Chl) index, the net photosynthesis rate (A), the stomatal conductance (g_s_), the intercellular CO_2_ concentration (C_i_), the intrinsic water use efficiency (WUE_i_) and the instantaneous carboxylation efficiency (A/C_i_) measured at the ‘VFB of the order 2 axes carried by the order 1 long axis in apical position’ stage for Knock Out^®^ Radrazz (KO) and ‘The Fairy’ (TF) grown in a climate chamber under two light conditions: WR and WRFR.

Genotype	Chl index (rel. units)		A (μmol CO_2_ m^−2^ s^−1^)		g_s_ (mol H_2_O m^−2^ s^−1^)		C_i_ (μmol CO_2_ mol^−1^)		WUE_i_		A/C_i_	
	WR	WRFR	WR	WRFR	WR	WRFR	WR	WRFR	WR	WRFR	WR	WRFR
KO	43.5b^1^	41.6a	7.0a	7.0a	0.26a	0.25a	330.1a	326.7a	33.0a	35.0a	0.021a	0.022a
TF	44.7b	39.5a	7.9a	8.6b	0.42a	0.37a	348.2a	341.8a	20.5a	24.5a	0.022a	0.025b

1 Means followed by the same lowercase letter in the same line are not significantly different (Tukey’s test, *p* < 0.05).

At biochemical level, a significant effect of FR was displayed in TF, with an increase in starch concentration in stem (+20.2%), while an increase in ABA concentration was observed both in stem (+7.3%) and leaves in KO (+37.4%; Table 6). For other sugar (sucrose and reducing sugars) and hormone (IAA, riboside zeatin, isopentenyladenosine, jasmonic acid and salicylic acid) concentrations measured both in stem and leaves, no significant effect of FR was displayed, whatever cultivar (Table 6).

**Table 6:** Least-square means (LS means) of the sugar and hormone concentrations in the stem and leaves of the order 1 long axis measured on the 19th day of cultivation for Knock Out^®^ Radrazz (KO) and ‘The Fairy’ (TF) grown in a climate chamber under two light conditions: WR and WRFR.

Genotype	Stem					
	Sucrose (mg/g DW)		Red. sugars (mg/g DW)		Starch (mg/g DW)	
	WR	WRFR	WR	WRFR	WR	WRFR
KO	22.2a^1^	22.1a	28.5a	27.7a	13.1a	13.5a
TF	16.3a	15.6a	13.3a	14.6a	10.4a	12.5b
Genotype	IAA (ng/g DW)		ABA (ng/g DW)		RZ (ng/g DW)	
	WR	WRFR	WR	WRFR	WR	WRFR
KO	103.7a	97.0a	2280.0a	2447.3b	8.6a	9.2a
TF	81.4a	87.0a	903.0a	902.5a	10.7a	14.4a
Genotype	I (ng/g DW)		SA (ng/g DW)		JA (ng/g DW)	
	WR	WRFR	WR	WRFR	WR	WRFR
KO	9.2a	9.1a	22023.9a	23569.6a	348.4a	432.9a
TF	3.7a	4.9a	10495.9a	14337.9a	482.2a	616.4a
Genotype	Leaf					
	Sucrose (mg/g DW)		Red. sugars (mg/g DW)		Starch (mg/g DW)	
	WR	WRFR	WR	WRFR	WR	WRFR
KO	54.6a^1^	50.7a	8.0a	7.1a	48.0a	49.2a
TF	46.4a	44.9a	3.5a	4.1a	23.9a	27.1a
Genotype	IAA (ng/g DW)		ABA (ng/g DW)		RZ (ng/g DW)	
	WR	WRFR	WR	WRFR	WR	WRFR
KO	25.0a	28.7a	672.3a	924.0b	1.6a	1.7a
TF	22.3a	19.9a	824.4a	877.1a	1.8a	2.1a
Genotype	I (ng/g DW)		SA (ng/g DW)		JA (ng/g DW)	
	WR	WRFR	WR	WRFR	WR	WRFR
KO	5.0a	4.7a	4779.7a	5342.4a	147.6a	183.0a
TF	2.7a	2.8a	7560.1a	7458.0a	147.0a	194.4a

Red. sugars, reducing sugars; IAA, indole-3-acetic acid; ABA, abscisic acid; RZ, riboside zeatin; I, isopentenyladenosine; SA, salicylic acid; JA, jasmonic acid.

1 Means followed by the same lowercase letter in the same line are not significantly different (Tukey’s test, *p* < 0.05).

## Discussion

Our findings demonstrate that rose is an interesting plant model to explore the basic mechanisms orchestrating the genotype-specific responses to FR. The two rose cultivars TF and KO exhibited fully contrasting architectural responses to FR that might be mainly assigned to sugar and ABA, two antagonistic endogenous growth factors in plant growth (Sakr et al., 2018). The architecture of TF proved very sensitive to FR, resulting in high elongation (+56.7% for LLA), branching (+24.7% for NbA) and accelerated flowering (+224.2% for NbDetA), in accordance with its responsiveness to light quality (Crespel et al., 2020). Interestingly, the effect of FR on TF differed from that reported in tomato (Kalaitzoglou et al., 2019; Zhang et al., 2019), soybean (Yang et al., 2020) or snapdragon (Park and Runkle, 2017), in which it was limited to axis elongation and flowering acceleration. FR-mediated branching has been reported in chrysanthemum, mainly limited to basal branching (Yuan et al., 2018), but nothing was said about other architectural traits. This highly architectural plasticity of TF to environmental conditions fits well with its responsiveness to light intensity and water stress (Crespel et al., 2014; Li-Marchetti et al., 2015). Unlike TF, KO was fully insensitive to light quality changes, confirming our previous observations regarding its insensitivity to variations in B/R and R/FR ratios (Abidi et al., 2013; Crespel et al., 2020). However, it was sensitive to light intensity (Demotes-Mainard et al., 2013; Corot et al., 2017).

The positive effect of FR on TF growth was associated with high biomass production (+26.7% for stem dry weight and + 25.0% for leaf dry weight) and the plant ability to intercept more light. This FR effect occurred through the increased total leaf area (+31.3%) and the lengthening of the order 1 long axis (+5.8%) that promoted better light distribution to the plant, as reported in geranium, snapdragon or tomato (Park and Runkle, 2017; Kalaitzoglou et al., 2019; Zhang et al., 2019). In TF, this better ability to intercept light was triggered as early as the 12th day of cultivation in the leaves that became more plagiotropic, and the 19th day for the order 1 long axis that grew longer and orthotropic. Although similar observations have been made in tomato (Kalaitzoglou et al., 2019) and soybean (Yang et al., 2020), the early regulatory mechanisms have not been addressed.

FR also increased the rate of net photosynthesis per unit area (+8.9%) in TF, as observed in lettuce (Zou et al., 2019), tomato (Kalaitzoglou et al., 2019) or soybean (Yang et al., 2020). However, this positive effect of FR on TF photosynthesis was neither associated with an increase in stomatal conductance, as observed in lettuce (Zou et al., 2019), nor with an increase in intercellular CO_2_ concentration. It might result from a more efficient use of the photons absorbed by photosystem II, thus leading to higher instantaneous carboxylation efficiency (+11.3%; Zhen and van Iersel, 2017). However, no significant effect of FR was displayed for intrinsic water use efficiency. A tendency to produce more biomass per unit of water used was nevertheless observed for FR. These two combined effects of FR on photosynthesis—at the plant scale and per unit area—accounted for the elevated starch concentrations in the stem (+20.2%) of TF. Extra FR also increased the soluble sugar and starch concentrations in the stems of tobacco, mustard or *Arabidopsis thaliana* (Kasperbauer et al., 1970; Casal et al., 1995; de Wit et al., 2018). However, this FR effect was not observed in KO. Thus, we assume that this high carbon status in TF serves as a driving force to support the FR-dependent stimulation of stem elongation, flowering and shoot branching. A great deal of research has demonstrated the key role of sugars in stem elongation and flowering, mainly through their interaction with hormones (Sakr et al., 2018; Gawarecka and Ahn, 2021). In addition, sugars play both trophic and signalling roles in shoot branching in a variety of species, including rose (Leduc et al., 2014; Rameau et al., 2015; Fichtner et al., 2017, 2021; Barbier et al., 2019, 2021; Schneider et al., 2019). As active sink organs, growing buds need to import sugar to meet the needs of their high metabolic activity (Girault et al., 2010; Henry et al., 2011; Rabot et al., 2012; Furet et al., 2014), involving remobilisation of stem-stored starch reserves (Alaoui Sosse et al., 1994; Maurel et al., 2004; Decourteix et al., 2008; Bonhomme et al., 2010; Girault et al., 2010; Barbier et al., 2015). Sugars also positively control bud outgrowth through their direct interaction with branching-related hormones (Barbier et al., 2015, 2021; Bertheloot et al., 2019; Wang et al., 2021). FR-mediated shoot branching, stem elongation and accelerated flowering might result from the elevated plant carbon status (sugar availability) and carbon allocation to the different sink organs (Schneider et al., 2019).

The photosynthetic capacities of KO, and therefore its carbon status, remained unchanged. In parallel, KO accumulated ABA in its leaves (+37.4%), and to a lesser extent in its stem (+7.3%) in response to FR, as previously reported in tomato (Tucker, 1977; Cagnola et al., 2012), sunflower (Kurepin et al., 2007) or chrysanthemum (Yuan et al., 2018). ABA is known to inhibit photosynthesis by limiting CO_2_ uptake through stomatal closure (Franks and Farquhar, 2001). The stomatal conductance nevertheless remained unchanged in response to FR. ABA is also known to inhibit photosynthesis by directly repressing photosynthesis-related genes (Acevedo-Hernández et al., 2005; Staneloni et al., 2008; Zhu et al., 2020; Müller and Munné-Bosch, 2021). Based on this, we speculate that FR-induced ABA accumulation in leaves might counteract the stimulating effect of FR on photosynthesis, and thereby on the overall plant carbon status required for stem elongation, shoot branching and flowering. ABA accumulates in the stem of KO at a low light intensity and accounts for branching inhibition (Corot et al., 2017). Decreased cytokinin and sugar concentrations were also observed in the stem, suggesting the involvement of two distinct mechanisms in the response of KO to light intensity and quality. One main future task will be to decipher the mechanisms behind FR-dependent ABA accumulation in the leaves.

## Conclusion

The effect of FR is genotype dependent in rose. This lays out the foundation for exploring the underlying mechanisms. The response of TF to FR was associated with an enhancement of its carbon status, while the insensitivity of KO might be assigned to ABA accumulation in its leaves. Further investigations will be set up to identify the molecular determinants of these contrasting responses. Regarding lighted horticultural crops, the addition of FR to R and B is widely used to increase agronomic performance in terms of flowers and fruit (Park and Runkle, 2017; Kalaitzoglou et al., 2019; Zhang et al., 2019). However, based on the genotype-specific response to light quality, special attention will be paid to the choice of cultivars grown in these lighted production systems.

## Data availability statement

The original contributions presented in the study are included in the article/supplementary material, further inquiries can be directed to the corresponding author.

## Author contributions

LC, CLB, TA, and SS designed the research. CLB, TA, BD, SC, and M-DP-G performed the experiments. LC and CLB analysed the data. LC and SS wrote the paper. All authors contributed to the article and approved the submitted version.

## Funding

This research work was financed by the French Ministry of Agriculture and Food (Compte d’Affectation Spéciale pour le Développement Agricole et Rural; CASDAR) within the framework of the IRRADIANCE project.

## Conflict of interest

The authors declare that the research was conducted in the absence of any commercial or financial relationships that could be construed as a potential conflict of interest.

## Publisher’s note

All claims expressed in this article are solely those of the authors and do not necessarily represent those of their affiliated organizations, or those of the publisher, the editors and the reviewers. Any product that may be evaluated in this article, or claim that may be made by its manufacturer, is not guaranteed or endorsed by the publisher.
